# Analysis of effects of a new environmental pollutant, bisphenol A, on antioxidant systems in soybean roots at different growth stages

**DOI:** 10.1038/srep23782

**Published:** 2016-03-31

**Authors:** Jiazhi Zhang, Xingyi Li, Li Zhou, Lihong Wang, Qing Zhou, Xiaohua Huang

**Affiliations:** 1 State Key Laboratory of Food Science and Technology, School of Environment and Civil Engineering, Jiangnan University, Wuxi 214122, China; 2 Jiangsu Collaborative Innovation Center of Biomedical Functional Materials, Jiangsu Key Laboratory of Biomedical Materials, College of Chemistry and Materials Science, Nanjing Normal University, Nanjing 210046, China; 3 Jiangsu Coorperative Innovation Center of Technology and Material of Water Treatment, Suzhou University of Science and Technology, Suzhou 215009, China

## Abstract

Bisphenol A (BPA) is an important industrial raw material. Because of its widespread use and increasing release into environment, BPA has become a new environmental pollutant. Previous studies about BPA’s effects in plants focus on a certain growth stage. However, the plant’s response to pollutants varies at different growth stages. Therefore, in this work, BPA’s effects in soybean roots at different growth stages were investigated by determining the reactive oxygen species levels, membrane lipid fatty acid composition, membrane lipid peroxidation, and antioxidant systems. The results showed that low-dose BPA exposure slightly caused membrane lipid peroxidation but didn’t activate antioxidant systems at the seedling stage, and this exposure did not affect above process at other growth stages; high-dose BPA increased reactive oxygen species levels and then caused membrane lipid peroxidation at all growth stages although it activated antioxidant systems, and these effects were weaker with prolonging the growth stages. The recovery degree after withdrawal of BPA exposure was negatively related to BPA dose, but was positively related to growth stage. Taken together, the effects of BPA on antioxidant systems in soybean roots were associated with BPA exposure dose and soybean growth stage.

Bisphenol A [BPA; 2,2-bis (4-hydroxyphenyl)] is a chemical intermediate for synthesizing polycarbonate and epoxy^1^. It is widely applied in the production of daily necessities, such as baby bottles, food packaging, thermal paper, electronic equipment and medical facilities^2^,^3^. Recently, it was reported that the global annual output of BPA is up to 6.8 million tons^4^. The production and consumption of BPA are fairly stable in Europe, but in Asia, especially China, they are increasing gradually^3^. Due to the massive use and emissions of BPA products, BPA is widespread in the global environment. It was reported that BPA concentrations in surface water in the Netherlands and Germany are up to 21 μg·L^−1 ^1 and 0.41 μg·L^−1 ^5, respectively. In Japan, the concentration of BPA in garbage leachate is 17.2 mg·L^−1 ^6. Toxicological studies on animals showed that BPA has estrogen activity that can disrupt physiological functions of animals and humans, especially in the reproductive and endocrine systems^7^,^8^,^9^.

Plants are the primary producers in ecological system, and can provide organic materials and energy for secondary consumers. Through food chains in ecological systems, the hazards of BPA can extend to animals, even humans^10^. The European Union has released a risk assessment report of BPA on terrestrial ecosystems. The draft of the report requires researchers to focus on BPA toxicity on plants to further investigate the potential risks of BPA on plants^11^. Recently, the effects of BPA on algae have been frequently reported^12^,^13^, but the effects of BPA on terrestrial plants are less studied^14^, and the microscopic mechanism of BPA on plants remains unclear^15^. Previous studies showed that certain doses of BPA exposure could promote or inhibit the growth^15^,^16^, germination^17^, pollen tube elongation^18^, photosynthesis^19^, and hormone contents^20^ in plants. As a vital organ of plants in long-term adaptations to land, roots can absorb water and nutrients from soil for plant growth and development^21^, and roots also have the ability to absorb, fix and transfer pollutants. Compared to the aerial parts of plants, roots are directly exposed to BPA in the soil; thus, it is important to study the microscopic mechanisms of BPA on plant roots. Previous studies showed that 1.5 mg·L^−1^ BPA could promote glutamine synthetase/glutamate synthetase cycles and glutamate dehydrogenase pathways in soybean roots for the synthesis of amino acids and proteins, thereby promoting root growth^22^. However, 17.2 and 50.0 mg·L^−1^ BPA inhibit glutamine synthetase/glutamate synthetase cycles and promote glutamate dehydrogenase pathways, leading to the production of excessive amino acids and proteins, thus inhibiting root growth^22^.

Reactive oxygen species (ROS) are a type of oxidants, which include superoxide anion (O_2_^−^), hydrogen peroxide (H_2_O_2_) and hydroxyl radical (·OH). They have active chemical properties and strong oxidizability^23^,^24^. ROS are produced in aerobic metabolism in plants^24^. As signaling molecules, ROS can regulate metabolism in plant roots, thus affecting plant growth and development^25^. Plants have formed complete antioxidant systems (antioxidant enzymes and non-enzymatic substances) during long-term evolution processes to regulate ROS levels and to resist adversity stress. The antioxidant enzymes mainly include superoxide dismutase (SOD), peroxidase (POD) and catalase (CAT)^26^, and antioxidant non-enzymatic substances mainly include glutathione (GSH), proline (Pro), and ascorbic acid (AsA)^27^. Therefore, studying the effects of BPA on ROS levels and antioxidant systems in plant roots is important for understanding root function, growth, and even the entire life status of plants.

Plant life cycles consist of different growth stages. The effects of the combined stress of soil acidification and lead ion on the antioxidant systems in soybean roots varies at different growth stages^28^. Unfortunately, the indicator systems used in previous studies on the environmental toxicology of BPA was relatively simple, or previous studies focused mainly on single growth stages in the plant life cycle^15^,^16^,^22^,^28^. Therefore, previous studies could not comprehensively, systematically, and accurately reflect plant responses to BPA exposure, thus the results could mislead scientists working to objectively evaluate the ecological risk of BPA. In this study, the effects of BPA on ROS levels, membrane lipid fatty acids, membrane lipid peroxidation, and antioxidant enzyme activities and non-enzymatic substance contents in soybean roots at different growth stages (seedling stage, flowering and podding stage, seed-filling stage) were investigated to evaluate whether there are differences among different growth stages. The results will provide new information to evaluate the microscopic mechanism of BPA on plants at all growth stages.

## Results

### Effects of BPA on ROS levels and membrane lipid peroxidation in soybean roots at different growth stages

**Figure 1:**
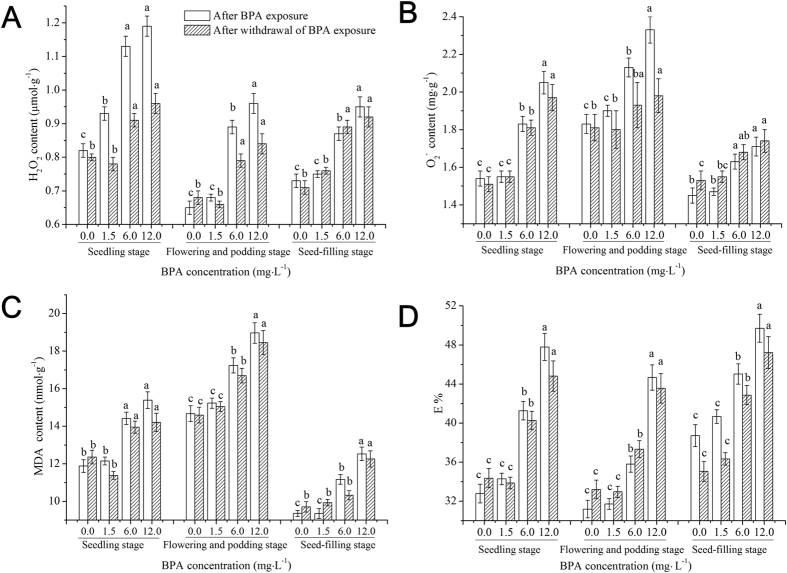
Effects of BPA on the contents of hydrogen peroxide (H_2_O_2_), superoxide anion (O_2_^−^), malondialdehyde (MDA) and membrane permeability (E%) in soybean roots at different growth stages following BPA exposure and withdrawal of BPA exposure. Significant differences at *p* < 0.05 are denoted with different letters at different growth stages.

Figure 1 shows the effects of BPA on ROS levels and membrane lipid peroxidation in soybean roots at different growth stages. Compared to the control, after 7 d of 1.5 mg·L^−1^ BPA exposure, the H_2_O_2_ content at the seedling stage increased (*p* < 0.05), but the O_2_^−^ and malondialdehyde (MDA) contents and membrane permeability did not change (*p* > 0.05), and those indices at both flowering and podding stages and seed-filling stages did not change (*p* > 0.05). With an increasing BPA dose (6.0 and 12.0 mg·L^−1^), the O_2_^−^ content, MDA content, and membrane permeability significantly increased (*p* < 0.05) at the three growth stages. Moreover, the increase degree of the O_2_^−^ content and membrane permeability were positively related to the BPA dose, but negatively related to the growth stage. Moreover, the O_2_^−^ content, MDA content, and membrane permeability at the three growth stages did not change (*p* > 0.05) after withdrawal of 1.5 mg·L^−1^ BPA exposure, but increased (*p* < 0.05) after withdrawal of 6.0 and 12.0 mg·L^−1^ BPA exposure.

### Effects of BPA on the composition of membrane lipid fatty acids in soybean roots at different growth stages

Tables 1, 2, and 3 show the effects of BPA on the composition of membrane lipid fatty acids in soybean roots at different growth stages. Compared to the control, after 7 d of 1.5 mg·L^−1^ BPA exposure, the composition of fatty acids at all growth stages did not change (*p* > 0.05), but the percentage content of different fatty acids changed (*p* < 0.05). The total contents of saturated fatty acids (SFA) increased (*p* < 0.05), whereas the total contents of unsaturated fatty acids (UFA) and the index of unsaturated fatty acids (IUFA) decreased (*p* < 0.05). After 7 d of 6.0 mg·L^−1^ BPA exposure, the SFA contents at the seedling stage as well as at the flowering and podding stage increased (*p* < 0.05), whereas the UFA content and IUFA decreased (*p* < 0.05). Moreover, only the IUFA at the seed-filling stage decreased (*p* < 0.05). After 12.0 mg·L^−1^ BPA exposure at different growth stages, the changes in all indices were similar to those after 6.0 mg·L^−1^ BPA exposure, and the change degrees were positively related to the BPA dose but negatively related to the growth stage. After withdrawal of BPA exposure, the SFA contents increased (*p* < 0.05) at the seedling stage, while the UFA content and IUFA decreased (*p* < 0.05); the SFA and UFA content as well as the IUFA at the flowering and podding stages did not change obviously (*p* > 0.05). Interestingly, the SFA content at the seed-filling stage decreased (*p* < 0.05).

### Effects of BPA on the activities of antioxidant enzymes in soybean roots at different growth stages

**Figure 2:**
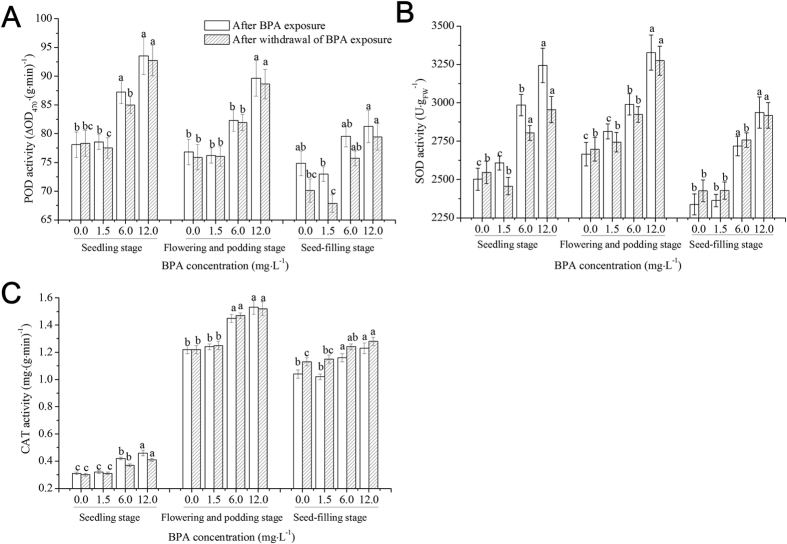
Effects of BPA on the activities of peroxidase (POD), superoxide dismutase (SOD) and catalase (CAT) in soybean roots at different growth stages following BPA exposure and withdrawal of BPA exposure. Significant differences at *p* < 0.05 are denoted with different letters at different growth stages.

Figure 2 shows the effects of BPA on the activities of antioxidant enzymes in soybean roots at different growth stages. Compared to the control, after 1.5 mg·L^−1^ BPA exposure, the activities of SOD, POD, and CAT at three growth stages did not change significantly (*p* > 0.05). After 6.0 mg·L^−1^ BPA exposure, the above indices increased (*p* < 0.05) at the seedling stage, and the SOD and CAT activities at the flowering and podding stage and the seed-filling stage increased (*p* < 0.05). After 12.0 mg·L^−1^ BPA exposure, the activities of the three antioxidant enzymes at the three growth stages increased significantly (*p* < 0.05). After withdrawal of 1.5 mg·L^−1^ BPA exposure, the activities of the antioxidant enzymes at different growth stages did not change (*p* > 0.05); however, after withdrawal of 6.0 and 12.0 mg·L^−1^ BPA exposures, the activities of antioxidant enzymes at all growth stages increased (*p* < 0.05), except for the POD activity at the flowering and podding stage after withdrawal of 6.0 mg·L^−1^ BPA exposure.

### Effects of BPA on the contents of antioxidant substances in soybean roots at different growth stages

**Figure 3:**
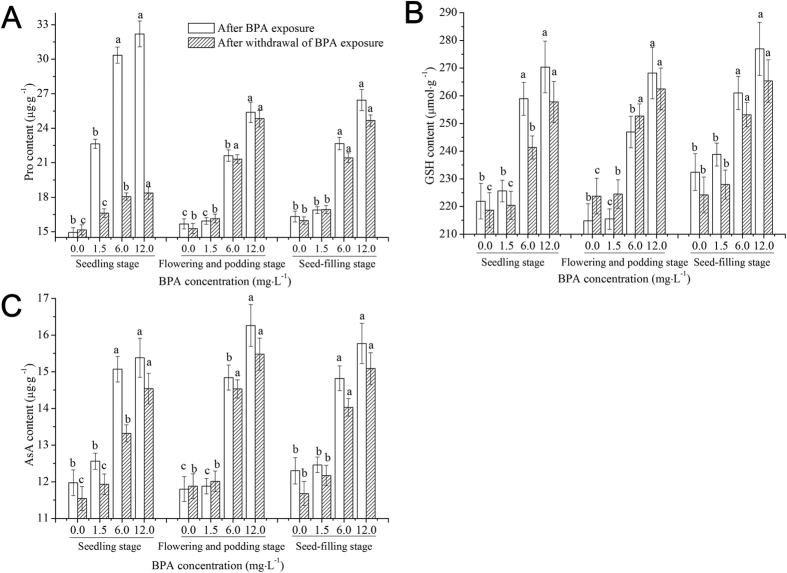
Effects of BPA on the contents of proline (Pro), glutathione (GSH) and ascorbic acid (AsA) in soybean roots at different growth stages following BPA exposure and withdrawal of BPA exposure. Significant differences at *p* < 0.05 are denoted with different letters at different growth stages.

Figure 3 shows the effects of BPA on the contents of antioxidant substances in soybean roots at different growth stages. Compared to the control, after 1.5 mg·L^−1^ BPA exposure, the Pro content at seedling stage increased (*p* < 0.05), and the contents of Pro, AsA, and GSH at the flowering and podding stage and the seed-filling stage did not change (*p* > 0.05). With an increase in the BPA dose (6.0 and 12.0 mg·L^−1^), the contents of all antioxidant substances at different growth stages increased (*p* < 0.05). After withdrawal of 1.5 mg·L^−1^ BPA exposure, the changes in the content of antioxidant substances at the seedling stage were similar with those after this dose of BPA exposure. The Pro content recovered, but did not recover to the control levels; however, the content of antioxidant substances at the latter two growth stages had no significant changes (*p* > 0.05). After withdrawal of 6.0 and 12.0 mg·L^−1^ BPA exposures, the content of the antioxidant substances at each growth stage increased (*p* < 0.05).

### Correlation analysis

Table 4 shows the relationship between antioxidant systems and ROS levels as well as membrane lipid peroxidation in soybean roots at different growth stages. At the seedling stage, after BPA exposure, H_2_O_2_ and MDA contents were positively correlated with antioxidant levels and O_2_^−^ content and membrane permeability were positively related to the activities of antioxidant enzymes and GSH content, but the IUFA showed no correlation with antioxidant levels. After withdrawal of BPA exposure, the correlations between ROS levels and antioxidant levels were positive, except for the Pro content; the MDA content was positively correlated with the SOD activity; and the IUFA was negatively correlated with the CAT activity and Pro content. At the flowering and podding stage, after BPA exposure, those correlation relationships were similar to those at the seedling stage, but the IUFA showed a negative correlation with most antioxidant system indexes. After withdrawal of BPA exposure, the correlations were similar to those after BPA exposure. At the seed-filling stage, after BPA exposure, the correlations became more significant than those at the flowering and podding stage, but the IUFA still showed no correlation with antioxidant system levels. After withdrawal of BPA exposure, the correlations were similar to those after BPA exposure, except that the MDA content showed no correlation with antioxidant levels.

## Discussion

The production and removal of ROS in plants maintain a dynamic balance under normal conditions^25^. Under adversity, however, a large number of ROS (H_2_O_2_ and O_2_^−^) will be produced in plant cells, thus breaking the balance^29^. Our results showed that low dose of BPA (1.5 mg·L^−1^) exposure caused higher production of H_2_O_2_ than removal in soybean roots (Fig. 1) at the seedling stage, and this imbalance was aggravated as BPA exposure dose increased (Fig. 1). After withdrawal of BPA exposure, the imbalance did not return to the control levels (Fig. 1). Excessive ROS can attack the polyunsaturated fatty acid in the cell membrane, causing membrane lipid peroxidation^30^ and an increase in membrane permeability. Moreover, excessive ROS can be used to generate more toxic ·OH by promoting the Fenton reaction^31^, affecting plant physiological activity and inhibiting plant growth and development^29^. MDA is a product of membrane lipid peroxidation; thus, the MDA content is an important index to evaluate the degree of membrane lipid peroxidation^32^. UFA is the main component of cell membrane lipids in organisms, and it can regulate a variety of abiotic stresses in plants^33^. Low dose of BPA (1.5 mg·L^−1^) exposure led to excessive production of ROS in soybean roots at the seedling stage, causing oxidative stress to the UFA (Table 1), rather than membrane lipid peroxidation (Fig. 1). Higher doses of BPA exposure (6.0 and 12.0 mg·L^−1^) caused excessive accumulation of ROS, leading to the self-catalytic effect of free radical chain reactions aggravating the oxidation of the UFA (Table 1), finally causing decreases in membrane fluidity and membrane lipid peroxidation (Fig. 1). SOD is the first line of defense in plants to disproportionate O_2_^−^ to H_2_O_2_. POD is an important enzyme in the cell walls or cytosol of plants, and it can catalyze reactions from H_2_O_2_ to H_2_O to achieve detoxification^34^. CAT is in the peroxisome or mitochondria, and its function is similar to that of POD, though CAT has a low substrate affinity^34^. AsA is an effective scavenger of free radicals and has antioxidant functions^35^, and the decrease in its levels indicates a decline in the total antioxidant capacity. As an antioxidant, GSH can directly remove ROS, and at the same time, GSH itself can be oxidized to oxidized glutathione^36^. Pro regulates the osmosis of cell membranes and coordinates intracellular metabolic processes^37^. In this work, we found that low dose (1.5 mg·L^−1^) of BPA exposure caused oxidative stress to soybean roots at the seedling stage (Fig. 1) and the accumulation of Pro in cells to regulate cell membrane permeability and reduce the absorption of BPA by roots^37^, but this did not cause membrane lipid peroxidation or activate the antioxidant systems (Figs 1, 2, 3). High doses of BPA exposure activated the antioxidant enzyme systems to remove excess ROS (Table 1), while membrane lipid peroxidation was not avoided (Fig. 1 and Table 1). After withdrawal of high doses of BPA exposure, membrane lipid peroxidation did not disappear, indicating that the structure of soybean roots at the seedling stage did not develop well and stress resistance and self-healing abilities were relatively low^38^; thus, BPA exposure damaged the membrane structure of soybean seedling roots.

To objectively evaluate whether the effects of BPA on antioxidant systems in soybean roots varies at different growth stages, we also studied the effects of BPA exposure on antioxidant systems in roots at the flowering and podding stage and the seed-filling stage. The flowering and podding stage is the important period for soybean to alternately grow and reproduce, as well as a period for forming dry matter. Our results showed that low-dose BPA (1.5 mg·L^−1^) exposure did not significantly affect ROS levels, membrane lipid peroxidation or the antioxidant systems in soybean roots at the flowering and podding stage (Figs 1, 2, 3 and Table 2). This result indicates that the growth and metabolism of soybean at the flowering and podding stage are relatively strong and able to accumulate more biomass, and the structure and function of roots are robust, which enables resilience to also be relatively strong^37^. The 6.0 mg·L^−1^ BPA exposure increased ROS levels, and the increased ROS attacked polyunsaturated fatty acids in roots’ cell membranes, causing decreases in the UFA content (Table 2). Meanwhile, the activities of antioxidant enzymes and the levels of non-enzymatic substances increased to remove excess ROS (Figs 2 and 3). However, the increased ROS in soybean root cells was beyond the elimination abilities of antioxidant enzymes and non-enzymatic substances, leading to excessive accumulation of ROS and membrane lipid peroxidation (Fig. 1)^30^. Moreover, after withdrawal of BPA exposure, membrane lipid peroxidation did not disappear (Fig. 1), indicating that high-dose BPA exposure caused irreversible damage to soybean roots at the flowering and podding stage. At the same time, the antioxidant ability and ROS levels were positively correlated (Table 4), indicating that high doses of BPA exposure did not cause fatal damage to the antioxidant systems of soybean roots.

The seed-filling stage is the key period for the yield formation of soybean^39^. Previous studies showed that the growth, photosynthesis, nitrogen transfer and assimilation of soybean declined after entering the seed-filling stage^40^, but soybean roots still showed higher activities at late growth stages^41^. Our data suggested that the effects of BPA exposure on ROS levels, membrane lipid peroxidation and antioxidant systems in soybean roots at the seed-filling stage were similar to those at the flowering and podding stage (Figs 1, 2, 3 and Table 3), and the H_2_O_2_ content was higher than the O_2_^−^ content (Fig. 1). We speculate that SOD constantly disproportionated O_2_^−^ to H_2_O_2_^42^ and that H_2_O_2_ is relatively stable and with greater longevity. Compared to the three growth stages, with the developing of soybean root structure and function, and the increasing of biomass, BPA stress on unit biomass of soybean roots was decreased^37^. Previous studies showed that low-dose (1.5 mg·L^−1^) BPA exposure can promote roots to absorb and make use of microelements (such as Mg and Mn)^43^, and microelements can be used to synthesize antioxidants to resist ROS oxidative stress^44^. The resistance of soybean roots to BPA stress at three growth stages followed the order: seed-filling stage > flowering and podding stage > seedling stage. The effects of BPA on antioxidant systems at different growth stages in the entire soybean plant, including leaves, should be an area of future investigation.

In conclusion, the effects of BPA on ROS levels, membrane lipid peroxidation and antioxidant systems in soybean roots were aggravated with increasing BPA exposure dose but weaker with prolonging the growth stages. After withdrawal of BPA exposure, these effects became weaker and the recovery degree was negatively related to BPA exposure dose and positively related to growth stage.

## Materials and Methods

### Preparation of BPA solution

Given current global BPA pollution situation, especially in developing countries^6^,^45^, previous studies about the effects of BPA on plants and animals^46^,^47^,^48^, and high environmental levels of BPA released by pollution accident, three BPA doses (1.5, 6.0, and 12.0 mg·L^−1^) were selected. Of these concentrations, 1.5 mg·L^−1^ BPA is assigned by the United States Environmental Protection Agency as a safe dose for drinking water and the upper safety limit for individuals^49^, and this dose is often used to investigate the effects of BPA on plants^17^,^47^,^49^; 6.0, and 12.0 mg·L^−1^ are the BPA concentrations in soil, river sediment, and hazardous landfill leachates^50^,^51^,^52^, and these two doses are also used to study the effects of BPA on plants^53^. Different doses of BPA solutions were prepared by dissolving suitable amounts of BPA in one-half strength Hoagland solution (pH 7.0).

### Plant culture and BPA exposure

Soybean (*Glycine max*, Zhonghuang 25) seeds were germinated in accordance with previously reported methods^19^. Thirty days after germination, when there were both flowers and young raised pods (approximately 2 cm) in the soybean plants, the soybean plants were transplanted into BPA solutions at different doses (0.0, 1.5, 6.0, and 12.0 mg·L^−1^). The control plants were cultured in one-half strength Hoagland nutrient solution (pH 7.0) without BPA. Each treatment was performed 6 times. The BPA solution and the nutrient solution were renewed every 3 d. The plants were exposed to BPA solution for 7 d and then moved to one-half strength Hoagland solution (pH 7.0) without BPA for 7 d, based on our previous pre-experiment (in which the results showed that the effect of BPA on soybean seedling growth became steady after BPA exposure for 7 d and withdrawal of BPA exposure for another 7 d). After 7 d of BPA exposure, followed by 7 d of BPA withdrawal, the roots were collected for measurements of test indices.

### Determination of ROS content and membrane lipid peroxidation

The H_2_O_2_ content in soybean roots was determined using a modification of a method described in previous reports^54^. Fresh roots (0.5 g) were homogenized in 3 mL of 50 mM potassium phosphate buffer (pH 6.5) at 4 °C and the homogenate was centrifuged at 11,500 × *g* for 15 min. The reaction mixture contained 3 mL supernatant and 1 mL of 20% H_2_SO_4_ (containing 0.1% TiCl_4_). The absorbance of the reaction mixture was determined at 410 nm, and the H_2_O_2_ content was calculated by a standard curve.

The O_2_^−^ content was determined according to previous methods^37^. Roots (2 g) were homogenized in 3 mL of 3% trichloroacetic acid, and then the homogenate was centrifuged at 12,000 × *g* for 15 min. The reaction mixture contained 1 mL supernatant and 1 mL of 50 mM potassium phosphate buffer (pH 7.0, containing 1 mM hydroxylamine hydrochloride). The absorbance of the reaction mixture was recorded at 530 nm, and the O_2_^−^ content was calculated by the standard curve.

The MDA content was determined according to previous reports^36^. Roots (0.5 g) were collected and homogenized in 3 mL 1% trichloroacetic acid, and the homogenate was centrifuged at 11,500 × *g* for 10 min. The reaction mixture contained 1 mL supernatant and 4 mL of 0.5% thiobarbituric acid, and it was reacted in a boiling water bath for 30 min then quickly cooled in an ice bath. The reaction mixture was centrifuged again at 11,500 × *g* for 15 min. The absorbance of the supernatant was recorded at 450 nm, 532 nm and 600 nm. The concentration of MDA was calculated according to the following equation: Concentration (μmol·L^−1^) = 6.45 × (OD_532_ − OD_600_)−0.56 × OD_450,_ where OD stands for optical density. The MDA content was expressed as nmol of MDA per g of fresh weight.

Membrane permeability was determined in accordance with previous methods, with slight modification^55^. Root fragments (0.5 g) were rinsed with deionized water and then put in glass tubes, which contained 10 mL deionized water. The roots were pumped in a vacuum environment for 20 min to translucent. The conductivity of the solution was measured with a conductivity meter (L_1_), and then the glass tubes were put in a boiling water bath to heat for 3–5 min. After being cooled to room temperature, the conductivity of the solution was measured again (L_2_). Membrane permeability was calculated using the followed formula: membrane permeability (E%) = (L_1_/L_2_) × 100%.

### Determination of fatty acid composition

The extraction of total membrane lipids in soybean root cells was based on previous reports^56^. Soybean roots (1.0 g) were baked at 105 °C for 5 min, then the samples were homogenized in a mixture of chloroform and methanol (1:2, V: V) and the homogenate was centrifuged at 1,000 × *g* for 10 min. The mixture of the supernatant and 0.76% NaCl was oscillated for 15 min and then concentrated by nitrogen rotary evaporator to obtain total membrane lipids. Fatty acid composition was determined by gas chromatography^57^. Methanol (2 mL) and concentrated sulfuric acid (4–5 drops) were added into the total membrane lipid. The mixture was put in a water bath (50–60 °C) for 10 min then added into n-hexane (1–2 mL). After shock, the mixture stood for 15 min, and 2 mL of distilled water were then added. The solvent in the supernatant was evaporated using nitrogen, after which the residues were analyzed using a Shimadzu GC-2010 gas chromatograph to automatically determine the sample. Standard substances of fatty acids were purchased from Sigma Company. The fatty acid content was obtained through comparisons with the peak area of the standard samples using quantitative analysis.

### Determination of antioxidant enzymes activity

Fresh soybean roots (0.5 g) were collected and homogenized in a 50 mM phosphate buffer (pH 7.8), which contained 5 mM ascorbic acid, 5 mM dithiothreitol, 5 mM EDTA, and 2% (v/v) polyvinylpyrrolidone. The homogenates were centrifuged at 15,000 × *g* for 15 min, and the supernatant was used to determine the antioxidant enzyme activity.

The SOD activity was determined using a modified method based on previous reports^36^. The mixture reacted under fluorescent lights for 30 min. One unit of enzyme activity was defined as the quantity of SOD required for 50% inhibition of nitroblue tetrazolium reduction at 560 nm.

The POD activity was determined in accordance with previously reported methods with slight modification^58^. The reaction mixture (3 mL) contained 1 mL 25 mM phosphate buffer (pH 6.8), 5 μL 28 mM guaiacol and 15 μL enzyme extract. A few drops of H_2_O_2_ were added to initiate reaction, and one unit of enzyme activity was defined as the change of absorbance at 470 nm within 1 min.

The determination of the CAT activity was based on previous reports^59^. The reaction mixture (3 mL) contained 100 mM phosphate buffer (pH 7.0) and 50 μL of enzyme extract. The 15 mM H_2_O_2_ (a few drops) was added into the mixture to initiate reaction. One unit of enzyme activity was defined as the decomposition of H_2_O_2_ (mg) from a fresh sample (g) at 240 nm within 1 min.

### Determination of the contents of non-enzymatic antioxidant substance

The AsA content was determined according to previous reports^37^. Roots (0.5 g) were homogenized in 5% trichloroacetic acid. Then, the homogenate was centrifuged at 4,000 × *g* for 10 min. The reaction mixture contained 0.2 mL supernatant, 0.5 mL of 150 mM phosphate buffer (pH 7.4, containing 5 mM EDTA) and 0.2 mL deionized water. The reaction mixture was incubated at 40 °C for 40 min and the absorbance was recorded at 532 nm. The AsA content was calculated by the standard curve.

The determination of the Pro content was carried out according to previous reports with slight modifications^36^. Roots (0.5 g) were collected and homogenized in 10 mL of 3% sulfosalicylic acid. Then, the homogenate was centrifuged at 1,000 × *g* for 10 min. The mixture (containing 2 mL supernatant, 2 mL ice acetic acid, and 4 mL 2.5% ninhydrin) underwent a reaction in a 100 °C water bath for 40 min and then was rapidly cooled to terminate the reaction. The absorbance was recorded at 520 nm and the Pro content was calculated by the standard curve.

The GSH content was determined in accordance with previously reported methods^36^. Roots (0.5 g) were collected and homogenized in 5 mL of 0.1 M HCl (pH 2.0), and the homogenate was centrifuged at 10,000 × *g* at 4 °C for 10 min. The reaction mixture contained 200 μL supernatant, 800 μL of 0.5 M KH_2_PO_4_/K_2_HPO_4_ buffer (pH 8.0), a few drops of 1 mM EDTA, and 100 μL of 6 mM 5, 5-dithiobis-(2-nitrobenzoic acid) (Sigma, USA). The mixture was reacted at 30 °C in a water bath for 15 min. The absorbance was determined at 412 nm, and the GSH content was calculated by standard curve.

### Statistical analysis

Each treatment group was set up 6 times, and all data for the three independent experiments of the mean value ± standard error (mean ± SD). The significant differences (*p* < 0.05) between the different treatments were analyzed using an LSD test with SPSS 17.0 software. The symmetric quantitative variables were used to calculate the Pearson’s correlation coefficient and to perform correlation analysis among test indices.

## Additional Information

**How to cite this article**: Zhang, J. *et al*. Analysis of effects of a new environmental pollutant, bisphenol A, on antioxidant systems in soybean roots at different growth stages. *Sci. Rep.* **6**, 23782; doi: 10.1038/srep23782 (2016).

## Figures and Tables

**Table 1:** Effects of BPA on the contents of saturated fatty acids (SFA), containing myristic acid (14:0), palmitic acid (16:0), stearic acid (18:0) and arachidic acid (20:0), the contents of unsaturated fatty acids (UFA), containing palmitoleic acid (16:1), oleic acid (18:1), linoleic acid (18:2) and linolenic acid (18:3) and the index of unsaturated fatty acids (IUFA) in the membrane lipid of soybean roots at the seedling stage following BPA exposure and withdrawal of BPA exposure.

BPA (mg·L^−1^)	Composition of fatty acids								SFA	UFA	IUFA
	14:00	16:00	16:01	18:00	18:01	18:02	18:03	20:00			
After BPA exposure											
0.0	0.58 ± 0.01c (100.00)	25.79 ± 0.42d (100.00)	0.63 ± 0.01b (100.00)	10.31 ± 0.17d (100.00)	2.71 ± 0.05a (100.00)	24.79 ± 0.40a (100.00)	33.01 ± 0.54a (100.00)	2.19 ± 0.04bc (100.00)	38.86 ± 0.63c (100.00)	61.14 ± 1.00a (100.00)	151.95 ± 2.47a (100.00)
1.5	0.80 ± 0.03b (137.93)	28.39 ± 0.98c (110.08)	0.62 ± 0.03b (98.41)	18.06 ± 0.62b (175.17)	2.19 ± 0.08b (80.81)	21.12 ± 0.73b (85.20)	26.12 ± 0.91b (79.13)	2.69 ± 0.09a (122.83)	49.95 ± 1.730b (128.54)	50.05 ± 1.73b (81.86)	123.42 ± 4.28b (81.22)
6.0	0.96 ± 0.02a (165.52)	31.78 ± 0.73b (123.23)	0.82 ± 0.04a (130.16)	15.69 ± 0.36c (152.18)	2.02 ± 0.06b (74.54)	22.26 ± 0.51b (89.79)	24.42 ± 0.57bc (73.98)	2.06 ± 0.05c (94.06)	50.49 ± 1.17b (129.93)	49.51 ± 1.14b (80.98)	120.60 ± 2.78b (79.37)
12.0	0.90 ± 0.03a (155.17)	35.94 ± 1.04a (139.36)	0.80 ± 0.02a (126.98)	23.33 ± 0.68a (226.29)	2.04 ± 0.05b (75.28)	15.42 ± 0.44c (62.20)	19.29 ± 0.55c (58.44)	2.28 ± 0.06b (104.11)	62.44 ± 1.80a (160.68)	37.56 ± 1.09c (61.43)	119.25 ± 3.44b (78.48)
After withdrawal of BPA exposure											
0.0	0.77 ± 0.02c (100.00)	27.95 ± 0.81c (100.00)	0.71 ± 0.02c (100.00)	10.86 ± 0.31b (100.00)	2.72 ± 0.08c (100.00)	25.18 ± 0.73a (100.00)	29.19 ± 0.84a (100.00)	2.61 ± 0.08b (100.00)	42.19 ± 1.22c (100.00)	57.81 ± 1.67a (100.00)	141.37 ± 4.08a (100.00)
1.5	0.83 ± 0.01bc (107.79)	31.86 ± 0.55b (113.99)	0.74 ± 0.01c (104.23)	11.48 ± 0.20b (105.71)	2.62 ± 0.05c (96.32)	22.54 ± 0.39b (89.52)	27.32 ± 0.47a (93.59)	2.61 ± 0.05b (100.00)	46.78 ± 0.81b (110.88)	53.22 ± 0.92b (92.06)	130.40 ± 2.26b (92.24)
6.0	0.91 ± 0.02b (118.18)	38.18 ± 0.88a (136.60)	1.00 ± 0.02b (140.85)	12.94 ± 0.30a (119.15)	3.39 ± 0.08b (124.63)	19.92 ± 0.46c (79.11)	20.01 ± 0.46b (68.55)	3.65 ± 0.10a (139.85)	55.69 ± 1.29a (132.00)	44.31 ± 1.02c (76.65)	104.25 ± 2.41c (73.74)
12.0	1.48 ± 0.04a (192.21)	37.10 ± 1.07a (132.74)	2.19 ± 0.06a (308.45)	13.62 ± 0.39a (125.41)	4.90 ± 0.14a (180.15)	17.20 ± 0.50d (68.31)	19.99 ± 0.58b (68.48)	3.52 ± 0.09a (134.87)	55.71 ± 1.61a (132.05)	44.29 ± 1.28c (76.61)	101.46 ± 2.93c (71.77)

Values are means ± standard deviation (*n* = 6). Values in parentheses are the percentage of treatments in control. Significant differences at *p* < 0.05 are shown with different letters in the same column following BPA exposure and withdrawal of BPA exposure.

**Table 2:** Effects of BPA on the contents of saturated fatty acids (SFA), containing myristic acid (14:0), palmitic acid (16:0), stearic acid (18:0) and arachidic acid (20:0), the contents of unsaturated fatty acids (UFA), containing palmitoleic acid (16:1), oleic acid (18:1), linoleic acid (18:2) and linolenic acid (18:3) and the index of unsaturated fatty acids (IUFA) in the membrane lipid of soybean roots at the flowering and podding stage following BPA exposure and withdrawal of BPA exposure.

BPA (mg·L^−1^)	Composition of fatty acids								SFA	UFA	IUFA
	14:00	16:00	16:01	18:00	18:01	18:02	18:03	20:00			
After BPA exposure											
0.0	0.68 ± 0.01b (100.00)	29.84 ± 0.49b (100.00)	1.07 ± 0.02a (100.00)	12.29 ± 0.20b (100.00)	4.96 ± 0.08a (100.00)	19.11 ± 0.31a (100.00)	29.75 ± 0.49a (100.00)	2.32 ± 0.04ab (100.00)	45.13 ± 0.74b (100.00)	54.87 ± 0.89ab (100.00)	133.47 ± 2.17ab (100.00)
1.5	0.60 ± 0.02c (88.24)	30.15 ± 1.05b (101.04)	0.92 ± 0.03b (85.98)	11.63 ± 0.40b (94.63)	3.36 ± 1.12b (67.74)	19.54 ± 0.68a (102.25)	31.69 ± 1.10a (106.52)	2.11 ± 0.08b (90.95)	44.49 ± 1.54b (98.58)	55.51 ± 1.92a (101.17)	138.43 ± 4.80a (103.72)
6.0	0.66 ± 0.02bc (97.06)	34.35 ± 0.79a (115.11)	0.82 ± 0.02c (76.64)	11.81 ± 0.27b (96.09)	3.43 ± 0.08b (69.15)	20.00 ± 0.46a (104.66)	26.47 ± 0.61b (88.97)	2.46 ± 0.06a (106.03)	49.28 ± 1.12a (109.20)	50.72 ± 1.17bc (92.44)	123.66 ± 2.86bc (92.65)
12.0	0.75 ± 0.02a (110.29)	33.52 ± 0.97a (112.33)	0.66 ± 0.02d (61.68)	14.15 ± 0.41a (115.13)	3.20 ± 0.09b (64.52)	20.50 ± 0.59a (107.27)	24.83 ± 0.72b (83.46)	2.38 ± 0.07a (102.59)	50.80 ± 1.47a (112.56)	49.20 ± 1.42c (89.67)	119.36 ± 3.45c (89.43)
After withdrawal of BPA exposure											
0.0	1.26 ± 0.03b (100.00)	34.61 ± 1.00ab (100.00)	1.48 ± 0.04a (100.00)	11.26 ± 0.32b (100.00)	5.69 ± 0.16b (100.00)	19.15 ± 0.55b (100.00)	23.17 ± 0.67a (100.00)	3.38 ± 0.10a (100.00)	50.51 ± 1.46ab (100.00)	49.49 ± 1.43ab (100.00)	114.98 ± 3.32a (100.00)
1.5	1.62 ± 0.03a (128.57)	36.61 ± 0.64a (105.78)	1.31 ± 0.02b (88.51)	11.67 ± 0.20b (103.64)	4.22 ± 0.08c (74.17)	19.80 ± 0.34ab (103.39)	22.21 ± 0.39a (95.86)	2.55 ± 0.05b (75.44)	48.46 ± 0.84b (95.94)	51.54 ± 0.89a (104.14)	119.84 ± 2.08a (104.23)
6.0	1.18 ± 0.03b (93.65)	37.13 ± 0.86a (107.28)	1.49 ± 0.03a (100.68)	12.68 ± 0.29a (112.61)	6.35 ± 0.14a (111.60)	18.51 ± 0.43b (96.66)	20.04 ± 0.46b (86.49)	2.62 ± 0.06b (77.51)	53.61 ± 1.24a (106.14)	46.39 ± 1.07b (93.74)	104.97 ± 2.42b (91.29)
12.0	0.87 ± 0.02c (69.05)	33.51 ± 0.97a (96.82)	1.01 ± 0.03c (68.24)	11.61 ± 0.33b (103.11)	5.72 ± 0.17b (100.53)	21.32 ± 0.62a (111.33)	23.49 ± 0.68a (101.38)	2.47 ± 0.07b (73.08)	52.44 ± 1.51ab (103.82)	47.56 ± 1.37b (96.10)	111.80 ± 3.23ab (97.23)

Values are means ± standard deviation (*n* = 6). Values in parentheses are the percentage of treatments in control. Significant differences at *p* < 0.05 are shown with different letters in the same column following BPA exposure and withdrawal of BPA exposure.

**Table 3:** Effects of BPA on the contents of saturated fatty acids (SFA), containing myristic acid (14:0), palmitic acid (16:0), stearic acid (18:0) and arachidic acid (20:0), the contents of unsaturated fatty acids (UFA), containing palmitoleic acid (16:1), oleic acid (18:1), linoleic acid (18:2) and linolenic acid (18:3) and the index of unsaturated fatty acids (IUFA) in the membrane lipid of soybean roots at the seed-filling stage following BPA exposure and withdrawal of BPA exposure.

BPA (mg·L^−1^)	Composition of fatty acids								SFA	UFA	IUFA
	14:00	16:00	16:01	18:00	18:01	18:02	18:03	20:00			
After BPA exposure											
0.0	1.64 ± 0.03c (100.00)	31.58 ± 0.51b (100.00)	2.28 ± 0.04d (100.00)	9.84 ± 0.16c (100.00)	6.91 ± 0.11c (100.00)	25.05 ± 0.41a (100.00)	19.26 ± 0.31a (100.00)	3.44 ± 0.06a (100.00)	46.50 ± 0.75a (100.00)	53.50 ± 0.87a (100.00)	117.08 ± 1.90a (100.00)
1.5	2.20 ± 0.08b (134.15)	34.81 ± 1.21a (110.23)	3.73 ± 0.13c (163.60)	11.96 ± 0.42a (121.54)	4.35 ± 0.15d (62.95)	22.37 ± 0.77b (89.30)	18.24 ± 0.63a (94.70)	2.34 ± 0.08c (68.02)	47.31 ± 1.64a (101.74)	52.69 ± 1.82a (98.49)	107.54 ± 3.72a (91.85)
6.0	2.95 ± 0.07a (179.88)	32.12 ± 0.74ab (101.71)	5.64 ± 0.12b (247.37)	10.41 ± 0.24bc (105.79)	12.48 ± 0.29a (180.61)	22.34 ± 0.51b (89.18)	11.11 ± 0.25b (57.68)	2.95 ± 0.07b (85.76)	48.43 ± 1.12a (104.15)	51.57 ± 1.19a (96.39)	96.13 ± 2.22b (82.11)
12.0	3.10 ± 0.09a (189.02)	33.49 ± 0.96ab (106.05)	4.21 ± 0.13a (184.65)	11.12 ± 0.32ab (113.01)	11.01 ± 0.32b (159.33)	22.35 ± 0.65b (89.22)	11.26 ± 0.32b (58.46)	3.45 ± 0.10a (100.29)	48.72 ± 1.48a (104.77)	48.83 ± 1.41a (91.27)	93.70 ± 2.70b (80.03)
After withdrawal of BPA exposure											
0.0	1.13 ± 0.03bc (100.00)	30.97 ± 0.89a (100.00)	1.43 ± 0.04c (100.00)	9.89 ± 0.28b (100.00)	4.28 ± 0.12a (100.00)	27.03 ± 0.78a (100.00)	22.75 ± 0.66a (100.00)	2.51 ± 0.08c (100.00)	44.51 ± 0.89a (100.00)	55.49 ± 1.60a (100.00)	128.02 ± 3.70a (100.00)
1.5	1.46 ± 0.02a (129.20)	29.62 ± 0.51a (95.64)	1.64 ± 0.03b (114.69)	9.16 ± 0.16c (92.62)	8.40 ± 0.14a (196.26)	25.28 ± 0.44b (93.53)	22.27 ± 0.39a (97.89)	2.17 ± 0.04d (86.45)	42.41 ± 0.85b (95.28)	57.59 ± 1.00a (103.78)	127.41 ± 2.21a (99.52)
6.0	1.11 ± 0.02c (98.23)	31.39 ± 0.73a (101.36)	1.51 ± 0.03bc (105.59)	11.45 ± 0.27a (115.77)	4.86 ± 0.11b (113.55)	24.13 ± 0.56b (89.27)	22.75 ± 0.53a (100.00)	2.81 ± 0.06b (111.95)	46.75 ± 0.94a (105.03)	53.25 ± 1.23a (95.96)	122.87 ± 2.83a (95.98)
12.0	1.21 ± 0.03b (107.08)	29.06 ± 0.84a (93.83)	3.38 ± 0.10a (236.36)	3.38 ± 0.10d (34.18)	3.38 ± 0.10d (78.97)	3.38 ± 0.10c (12.50)	3.38 ± 0.10b (14.86)	3.38 ± 0.10a (134.66)	37.48 ± 0.74c (84.21)	53.52 ± 1.55a (96.45)	123.66 ± 3.57a (96.59)

Values are means ± standard deviation (*n* = 6). Values in parentheses are the percentage of treatments in control. Significant differences at *p* < 0.05 are shown with different letters in the same column following BPA exposure and withdrawal of BPA exposure.

**Table 4:** Relationships between antioxidant systems and ROS levels as well as membrane lipid peroxidation in soybean roots at different growth stages following BPA exposure and withdrawal of BPA exposure.

	SOD activity	POD activity	CAT activity	AsA content	Pro content	GSH content		SOD activity	POD activity	CAT activity	AsA content	Pro content	GSH content
	After BPA exposure							After withdrawal of BPA exposure					
Seedling stage													
H_2_O_2_ content	0.977^*^	0.952^*^	0.976^*^	0.990^*^	0.989^*^	0.979^*^	H_2_O_2_ content	0.997^**^	0.974^*^	0. 982^*^	0.969^*^	0.863	0.983^*^
O_2_^−^ content	0.992^**^	1.000^**^	0.988^*^	0.948	0.890	0.983^*^	O_2_^−^ content	0.972^*^	0.980^*^	1.000^**^	0.997^**^	0.922	0.997^**^
MDA content	0.995^**^	0.992^**^	0.999^**^	0.999^**^	0.987^**^	0.963^*^	MDA content	0.973^*^	0.898	0.901	0.873	0.753	0.903
E%	0.996^**^	0.997^**^	0.984^*^	0.948	0.910	0.980^*^	E%	0.989^*^	0.995^**^	0.992^**^	0.988^*^	0.865	0.997^**^
IUFA	−0.728	−0.653	−0.692	−0.759	−0.905	−0.701	IUFA	−0.902	−0.884	−0. 956^*^	−0.946	−0.982^*^	−0.936
Flowering and podding stage													
H_2_O_2_ content	0.939	0.945	0. 999^**^	0. 995^**^	0.988^*^	0.986^*^	H_2_O_2_ content	0.930	0.968^*^	0. 979^*^	0.992^**^	0.981^*^	0.993^**^
O_2_^−^ content	0.985^*^	0.978^*^	0.985^*^	0.988^*^	0.994^**^	0. 993^**^	O_2_^−^ content	0.934	0.970^*^	0.987^*^	0.997^**^	0.988^*^	0. 998^**^
MDA content	0.985^*^	0.979^*^	0.986^*^	0.989^*^	0.995^**^	0.994^**^	MDA content	0.988^*^	0.994^**^	0.958^*^	0.975^*^	0. 995^**^	0.972^*^
E%	0.979^*^	0.990^*^	0.919	0.937	0.959^*^	0.961^*^	E%	0.995^**^	0.997^**^	0.915	0.947	0.972^*^	0.944
IUFA	−0.856	−0.940	−0.959^*^	−0.968^*^	−0.960^*^	−0.962^*^	IUFA	−0.433	−0.593	0.740	−0.711	−0.622	−0.723
Seed-filling stage													
H_2_O_2_ content	0.999^**^	0.953^*^	0.987^*^	0.995^**^	1.000^**^	0.999^**^	H_2_O_2_ content	0.970^*^	0.914	0.990^**^	0.986^*^	0.973^*^	0.984^*^
O_2_^−^ content	0.998^**^	0.963^*^	0.989^*^	0.999^**^	0.998^**^	0.996^**^	O_2_^−^ content	0.997^**^	0.966^*^	0. 999^**^	0. 998^**^	0.996^**^	1.000^**^
MDA content	0.998^**^	0.965^*^	0.994^**^	0.989^*^	0.998^**^	0.991^**^	MDA content	0.886	0.871	0.871	0.892	0.917	0.882
E%	0.988^*^	0.912	0. 963^*^	0.976^*^	0.990^**^	0.996^**^	E%	0.995^**^	0.964^*^	0.996^**^	0.999^**^	1.000^**^	0.998^**^
IUFA	−0.924	−0.840	−0.883	−0.938	−0.926	−0.948	IUFA	−0.919	−0.877	−0.938	−0.921	−0.897	−0.929

^*^p < 0.05; ^**^p < 0.01.
